# Physicochemical and Functional Modifications of Hemp Protein Concentrate by the Application of Ultrasonication and pH Shifting Treatments

**DOI:** 10.3390/foods11040587

**Published:** 2022-02-18

**Authors:** Ozan Kahraman, Greg E. Petersen, Christine Fields

**Affiliations:** Applied Food Sciences Inc., 8708 S. Congress Avenue STE B290, Austin, TX 78745, USA; gpetersen@appliedfoods.com (G.E.P.); cfields@appliedfoods.com (C.F.)

**Keywords:** hemp protein, ultrasound, pH shifting, SDS-PAGE, free sulfhydryl group

## Abstract

According to the Food and Agriculture Organization (FAO), protein demand is expected to increase globally by around 40% by 2030 as a response to the world’s population growth. Due to their clean label, vegan or vegetarian based applications, nutritional value, and cost-efficient properties, plant-based proteins have been widely studied. However, most of the alternatives currently found in the market have some challenges because of their poor solubility, emulsifying, gelling, and foaming attributes. Hemp seed protein has gained increasing attention due to its unique amino acids and fatty acids profiles. In this study, commercial HPC mixtures were adjusted to pH 2, 4, 6, 8, 10, and 12 followed by ultrasonication (US) for 5 min (5 s on: 5 s off) and incubated for an hour before neutralizing to pH 7. Following the treatments, the samples were analyzed for their hydrodynamic diameter, conductivity, zeta potential, polydispersity index, surface hydrophobicity, solubility, electrophoresis (SDS-PAGE), free sulfhydryl group, and optical characteristics. The samples treated with ultrasound at pH 8 and 10 significantly (*p* < 0.05) enhanced the solubility of the hemp seed protein by 12.12% and 19.05%, respectively. Similarly, the samples treated with ultrasonication and pH shifting at pH 6, 8, and 10 also significantly increased the amount of free sulfhydryl content (*p* < 0.05) to 41.6, 58.72, and 46.54 mmol/g from 32.8 mmol/g, respectively. This study shows that the application of ultrasonication and pH shifting is a promising alternative method to modify the functional properties of HPC and widen their applications in the food, cosmetics, and pharmaceutical industries.

## 1. Introduction

Proteins have a fundamental role both in sustaining human life and food products and they can be derived from animals or plants. They are structural macromolecules accounting for almost 75% (d.b.) of total body weight which are broken down to amino acids and used to synthesize hormones and enzymes, form bones, support the immune system, and build other functional compounds, such as antibodies [1]. In addition to their important roles in health and nutrition, proteins have many functional properties (water holding, emulsifying, foaming, gelation, etc.) which make them useful as multipurpose ingredients for many applications and/or formulations in food, animal feed, pharmaceutical, and chemical industries [2].

According to the Food and Agriculture Organization (FAO), the world population is projected to increase almost 70% by 2050 and this growing population will result in an increased demand for protein. It is expected that animal and plant sourced protein demand globally will increase 33 and 43%, respectively [3]. The awareness about the challenge of the growing world population encourages scientists to search for sustainable and economically feasible alternatives for animal-based proteins. It has been understood that livestock farming, and hence animal-based products have a serious impact on today’s environmental problems, such as climate change, greenhouse gas emissions, biodiversity loss, and pollution, which make them unsustainable and impractical [4]. Therefore, recently, plant-based proteins are increasingly being used as an alternative protein source in different industries. There are many oil crops that can be used as protein sources due to their high protein content, such as soybean, canola, flax seed, cotton seed, and sunflower. Hemp seeds have gained a growing interest in the food industry because of its rich nutrient composition and functionality. It is harvested worldwide and is economical due to its lower water consumption and easy cultivation compared to other plants. It is also known as a renewable energy source because of its high biomass and energy concentration. Hemp seeds mainly consist of 30–35% oil, 20–25% protein, and 25–30% carbohydrates. Due to its essential fiber, amino acid, and unsaturated fatty acid contents, hemp seed carries a great potential to be an alternative protein source for human consumption [5]. Previous studies reported that hemp protein contains all nine essential amino acids and has a comparable amino acid profile to well-known high-quality proteins, such as casein, soy protein, and egg white protein [6]. It has been also shown that hemp protein is abundant in glutamic acid and arginine, which are not commonly found in other oil seed proteins. Arginine has a considerable role in ammonia detoxification, fetal growth, and the maintenance of normal blood pressure, while glutamic acid has a vital role as a neurotransmitter in the brain [7,8]. The amino acid score for hemp protein is between 0.5 and 0.62, with lysine being the first limiting amino acid, followed by leucine and tryptophan [9,10]. Moreover, compared to cereal grains such as whole wheat, hemp protein has a higher protein digestibility-corrected amino acid score (PDCAAS) value, which is close to the values of some legume proteins, such as beans and lentils [9].

Despite its nutritious composition, hemp seed protein has a limited utilization in the food and pharmaceutical industries due to its poor functional properties, which is a common problem in many plant-based proteins. Various physical, chemical, and enzymatic processing methods have been employed to overcome these poor functional properties [11,12,13]. Ultrasonication is a novel processing technology which can ameliorate the functional deficiencies of hemp protein by modifying its structural properties. The application of power ultrasonication leads to different cavitation activities, including shock waves, shear forces, and localized high pressure and temperature in the medium [14]. This phenomenon contributes to the modification of the structural and functional properties of proteins, such as particle size, surface hydrophobicity, solubility, etc. [15,16,17,18,19,20]. A recent systematic review study also revealed that ultrasonication treatment improved not only the solubility, but also the emulsifying activity, foam stability, and foaming capacity of different plant-based proteins [21]. In addition to ultrasonication treatment, pH shifting is another process that could affect the protein structure by folding and unfolding at different pH levels. Previous studies have declared that pH shifting treatments could alter the globular structure of proteins, resulting in an increased amphipathic property of the proteins [20,22,23]. There are also several studies in which pH shifting was used as a pretreatment process or combined with ultrasonication in order to advance the functional properties of plant-based proteins such as soy, pea, and rice protein [22,24,25,26].

In this study, the main objective was to investigate the effects of ultrasonication combined with pH shifting treatments on the physicochemical properties of hemp protein concentrate. The possible outcome of this research can provide a potential solution to improve and modify the functional attributes of hemp protein concentrate which would increase its application in a broad range of fields, including food, cosmetics, and pharmaceutical industries.

## 2. Materials and Methods

### 2.1. Materials

Hemp seed protein concentrate samples (76% hemp protein (d.m.)) were provided by Applied Food Sciences Inc. (Austin, TX, USA). This commercial HPC was chosen as the starting medium for the experiments and stored at room temperature before use. All other chemicals were purchased from Sigma-Aldrich (St. Louis, MO, USA), Fisher Scientific (Pittsburgh, PA, USA), and Bio-Rad (Hercules, CA, USA). All chemicals used in this study were analytical or higher grade.

### 2.2. pH Shifting and Ultrasonication Treatments

The HPC dispersions were prepared by dissolving 6 g of HPC in 200 mL of deionized water in a 250 mL beaker and mixed for 30 min at room temperature with a magnetic stirrer, followed by homogenization for 5 min at 10,000 rpm (Model 850, Thermo Fisher Scientific, Waltham, MA, USA). Three treatments were applied to HPC dispersions: pH shifting alone, ultrasonication alone, and ultrasonication and pH shifting combined. The non-treated but only stirred sample was used as the control.

The ultrasonication and pH shifting treatments were applied to HPC dispersions following the methods of Lee et al. [24] and Jiang et al. [26] with slight modifications. A VibraCell VC750 ultrasonic processor (Sonics & Materials, Inc., Newtown, CT, USA) was used to apply ultrasonication treatment at 20 kHz and 750 W. The acoustic energy was transmitted into HPC dispersions using a 13 mm diameter probe. An ice bath was utilized to keep the sample temperature below 40 °C and to prevent possible overheating due to ultrasonication that could lead to protein denaturation. Duty cycle of sonication pulse was set at 100% amplitude (5 s on: 5 s off). During the ultrasonication alone treatment, the HPC dispersions were sonicated for 5 min, and then were kept at room temperature for an hour. In the pH shifting alone treatments, the pH of the HPC dispersion samples were adjusted to pH 2, 4, 6, 8, 10, or 12 with 1 M NaOH or 1 M HCl at room temperature. Following the one-hour incubation at room temperature, their pH was adjusted back to pH 7. Finally, in the ultrasonication and pH shifting combined treatments, the HPC dispersion samples were exposed to sonication for 5 min immediately after they were treated with pH shifting. All the treated samples were finally centrifuged at 12,400× *g* at 20 °C for 20 min (Sorvall Legend XTR centrifuge, Thermo Scientific, Osterode am Harz, Germany). The supernatants were collected for further analysis.

### 2.3. Acoustic Energy Determination

As ultrasound goes through the liquid matrix, some of the sonication power transforms into heat energy. The amount of lost acoustic power (power loss) was calculated as described in the study of Guzel et al. [27]. The temperature was recorded during the first 30 s. The rate of temperature increase was then calculated from the slope of temperature versus time graph created using the recorded temperature data. The power loss was calculated by using Equation (1):

(1) $$P=mC_{p}dT/dt$$

where *m* is the mass of the liquid used (g) and *Cp* is the heat capacity of the liquid (in J g^−1^ °C^−1^).

The unit of power loss is watts per unit volume of the sonicated solution (W/cm^3^). The determined lost sonication power in the liquid was 13.25 ± 0.18 W.

### 2.4. Hydrodynamic Diameter, Conductivity, Zeta-Potential, and Polydispersity Index

The hydrodynamic diameter (µm), conductivity (mS/cm), zeta potential (mV), and polydispersity index (%) of the HPC dispersions were measured by dynamic light scattering (DLS) and electrophoretic light scattering (ELS) (Litesizer 500, Anton Paar, Graz, Austria) with a 40-mW semiconductor laser with a wavelength of 658 nm, and the detection angle of back (175°), side (90°), and forward (15°).

The random movement and speed of particles in a liquid medium depends on particle size. The DLS method measures this constant movement of suspended particles in a solution. This movement or motion is also known as Brownian movement/motion [28]. In this research, the hydrodynamic diameter and polydispersity index were measured using a 2 mL disposable cuvette at 25 °C. 

The zeta potential of HPC dispersions was analyzed using the Kalliope 2.2 software at 25 ± 0.1 °C with an equilibration time of 1 min. An amount of 1 mL of each sample was placed in an omega cuvette without any air bubbles. Three replicates were carried out for each sample, with 100 scans per individual measurement.

### 2.5. Optical Characterizations and Turbidity

The turbidity of the HPC samples was carried out on a Cytation 5 multimode microplate reader (BioTek Instruments, Inc., Winooski, VT, USA), using 96-well polystyrene microplates. Distilled water was used as the blank and the absorbance was measured at 600 nm. The average of the three replications were reported.

Changes in the color of treated and untreated HPC dispersions were monitored with a Vista colorimeter (HunterLab, VA, USA) based on the CIE L*, a*, and b* color coordinates. The L* indicates brightness/darkness index (0 to 100/black to white), a* indicates redness/greenness (‘+’ values for red, ‘−’ values for green), and b* indicates yellowness/blueness (‘+’ values for yellow, ‘−’ values for blue). An amount of 2 mL of sample was placed in a disposable cuvette and three-color readings (L*, a*, and b* values) per sample were taken at room temperature. The average L*, a*, and b* values were reported. These values were used to calculate the following color parameters:

Total color change (Δ*E*) represents the level of total color alteration between the initial and final samples. Equation (2) was used to calculate the total color change (Δ*E*) of the dispersions:

(2) $$\Delta E=\sqrt{{\left({\Delta L}^{*}\right)}^{2\;}+{\left({\Delta a}^{*}\right)}^{2\;}+{\left({\Delta b}^{*}\right)}^{2}}$$

where ΔL*, Δa*, and Δb* were the difference of individual L*, a*, b* color readings from the control sample [29].

### 2.6. Surface Hydrophobicity Determination

Surface hydrophobicity was measured following the method described by Kato and Nakai [30] and Wang et al. [11] with slight modifications. Briefly, 1-Anilino-8-naphthalene sulphonate (ANS) (Sigma-Aldrich, St. Louis, MO, USA) was utilized as the fluorescence probe. An 8 mM ANS stock solution was prepared in 0.01 M, pH 7 phosphate buffer. Each HPC sample was diluted to five series of concentrations ranging from 0.025 to 0.2 mg/mL. Then, 20 µL of ANS was added into 4 mL of sample. The fluorescence intensity was measured (excitation and emission were 340 nm and 440 nm, respectively) with a Cytation 5 multimode microplate reader (BioTek Instruments, Inc., Winooski, VT, USA). The initial slope of fluorescence intensity versus protein concentration (mg/mL) plot of the serial dilutions were used to calculate the surface hydrophobicity.

### 2.7. Protein Quantification (Solubility) and Electrophoresis (SDS—PAGE)

The solubility of the HPC samples was determined with the Pierce BCA Protein Assay Kit (Thermo Scientific) based on the bicinchoninic acid (BCA) method using bovine serum albumin (BSA) as the standard. The samples were centrifuged at 12,000× *g* for 15 min, and the supernatant was collected and diluted 1:10 with water. The standard curve was prepared using 2 mg/mL BSA stock solution at different concentrations levels (50 µg/mL–400 µg/mL). After that, 25 µL standards and/or samples were loaded in triplicate into a 96 well microtiter plate and 200 µL BCA reagent (50 parts BCA reagent A to 1-part BCA reagent B) was added to each well. The microplate was mixed and incubated at 37 °C for 30 min. Following incubation, the plate was allowed to cool for approximately 10 min, then the absorbance was read at 562 nm with a multimode microplate reader (Cytation 5, BioTek Instruments, Inc., Winooski, VT, USA). The protein concentrations were calculated using the BSA standard curve (R^2^ = 0.996). The amount of soluble protein was represented as mg/mL.

The samples were evaluated by SDS-PAGE, as described by Tang et al. [6] with modifications, on 8–16% precast gels (Mini-Protean TGX Stain-Free Protein Gel, Bio-Rad Laboratories, Hercules, CA, USA) using a Mini Protean III Electrophoresis Cell (Bio-Rad Laboratories, Hercules, CA, USA). The samples were diluted (1:30 *v*/*v*) in 1X Laemmli sample buffer (Bio-Rad Laboratories, Hercules, CA, USA) in the presence (reducing condition) or absence (non-reducing condition) of 50 mM DTT and denatured at 95 °C for 5 min. The samples (4 µL) and markers (4 µL) were loaded on the gel and electrophoresed at a constant voltage (145 V) for approximately 50 min at room temperature. Following separation, the gels were stained with Coomassie Brilliant Blue R-250 Staining Solution (Bio-Rad Laboratories, Hercules, CA, USA) overnight and destained using reverse osmosis water. The gels were imaged using a Bio-Rad ChemiDoc MP (Bio-Rad Laboratories, Hercules, CA, USA).

### 2.8. Free Sulfhydryl Group Analysis

The free sulfhydryl group content of the samples was analyzed following the method of Beveridge et al. [31] and manufacturer’s instructions with some modifications. 5,5′-Dithio-bis-(2-Nitrobenzoic Acid) (DTNB) and Ellman’s reagent (Thermo Fisher Scientific, Waltham, MA, USA) were utilized to determine the content of the free sulfhydryl group in the samples. Sodium phosphate buffer (0.1 M, pH 8.0) was used to dilute the protein solution to a certain concentration. l-Cysteine hydrochloride (Alfa Aesar, Tewksbury, MA, USA) was used as a standard. Dilutions of cysteine (0.25–1.5 mM) sodium phosphate buffer (0.1 M, pH 8.0) were prepared to plot a standard curve. An amount of 50 μL of Ellman’s reagent solution (4 mg/mL) was added in the mixture of 250 μL of protein sample and 2.5 mL of sodium phosphate buffer. After incubation at room temperature for 15 min, the absorbance was measured in triplicates at 412 nm using a multimode microplate reader (Cytation 5, BioTek Instruments, Inc., Winooski, VT, USA). The free sulfhydryl group content was expressed as μmol/g protein.

### 2.9. Statistical Analysis

All experiments were conducted in triplicates. The results were expressed as mean value ± standard deviation (S.D.). Rstudio software was used to conduct the statistical analysis of the collected data. Differences between the treatments were assessed by running One-way ANOVA, followed by conducting Fisher’s least significant difference (LSD) test. Significant differences among the samples were detected at *p* <0.05.

## 3. Results and Discussion 

### 3.1. Hydrodynamic Diameter, Zeta-Potential, Conductivity and Polydispersity Index

The hydrodynamic diameter of proteins has a crucial effect on the functional and physical properties of proteins, such as the solubility, emulsification, foaming capacity, and gel properties [32]. The hydrodynamic diameter, conductivity, and polydispersity index of treated and non-treated HPC dispersions are shown in Table 1. According to the results, after the combined treatment of pH shifting at pH 4 and ultrasonication, the particle size of HPC dispersions decreased significantly (*p* < 0.05) from 1.05 ± 0.05 µm to 0.33 ± 0.02 µm, and their polydispersity index values also showed a significant reduction (*p* < 0.05) from 55.9 ± 1.2% to 12.1 ± 0.02%. Moreover, regardless of targeted pH levels, combining pH shifting with ultrasonication resulted in lower hydrodynamic diameter than control and pH shifting alone treatments. HPC dispersions at different pH alone treatments unfolds and partially disaggregates, while application of ultrasonication may further disrupt the interactions between protein molecules by ultrasonic physical forces (shear forces, turbulent forces, cavitational forces, etc.) [17,19,33] and escalate these changes. Therefore, the hydrodynamic diameter of samples treated with the combination of pH shifting and ultrasonication treatments had smaller particle size than the non-sonicated samples. These results agree with earlier studies. In the study of Jiang et al. [25], the particle size of pea protein isolate treated with ultrasonication was two times smaller than that of the control. Similarly, the application of ultrasonication and pH shifting significantly reduced (*p* < 0.05) the particle size of rice protein (219.8–248.8 nm) compared to the control (486.4 nm) [34]. 

With respect to polydispersity index, all ultrasonication treated HPC dispersions (ranging from 12.10 to 17.30%) showed significantly lower values than the control (55.90%) and pH alone treated dispersions (ranging from 40.88 to 46.80%). Zhang et al. [34] also reported that the polydispersity of rice protein was reduced almost by 40% with the application of sonication at 20 kHz frequency for 60 min at 50 °C. This might be related to the intensification of the cavitation and mechanical effects of ultrasonication treatments [20]. The reduction in particle size can enhance the interaction between water and protein molecules leading to an increase in solubility [12,35,36].

**Figure 1:**
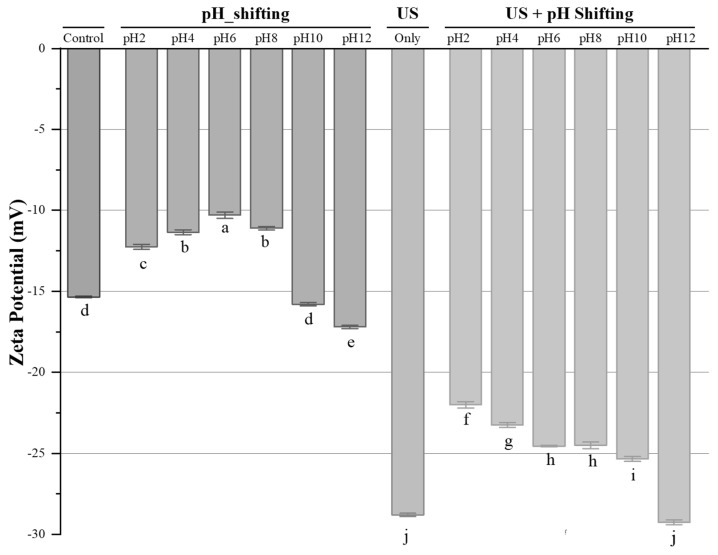
The zetapotential of non-treated and treated HPC samples. ^a–j^ Treatment means with the same letter in each sample are not significantly different (*p* > 0.05).

Zeta potential is widely used to characterize and measure the surface charges of protein molecules and the strength of repulsion or attraction between particles based on electrophoretic mobility technique [18]. As large repulsive forces between particles result in high absolute zeta potential values, a more stable system can be achieved with small, dispersed particles [34]. As it can be seen in Figure 1, the absolute value of zeta potential increased with an increasing pH level both in pH shifting alone and pH shifting with ultrasonication treatments, except at acidic pH levels during pH shifting alone treatments. The absolute zeta potential value of ultrasonication treated HPC dispersion samples raised significantly (*p* < 0.05) from [15.4 ± 1.28] mV to [29.4 ± 1.41] mV compared to the control. This might be explained with the cavitation effect of ultrasonication on the protein structure, which causes breakdowns and allows more charges on the molecules [32]. Similarly, Shen et al. [18] observed that the application of ultrasonication significantly increased (*p* < 0.05) the absolute zeta potential of whey proteins from 14.9 mV to 27.94 mV at 20 kHz frequency for 20 min (10 s:5 s off/on cycles). On the other hand, the decreasing trend in absolute zeta potential values at the acidic pH levels during pH shifting alone treatments might be because of the change in ionization degree of the surface groups in HPC dispersions [32]. 

The conductivity of protein is an inherent property of dissolved protein particles which depends on the amount of charged ions [17]. In the present study, the conductivity of HPC dispersions did not change significantly (*p* > 0.05) after pH alone treatments. However, when ultrasonication treatment was applied, the conductivity of HPC samples increased from 0.96 ± 0.004 mS/cm to 2.962 ± 0.01 mS/cm. Jambrak et al. [15,37] also affirmed that ultrasonication can increase the conductivity of whey protein suspensions and soy proteins. This increasing trend might be explained by the formation of radicals due to the localized extreme physical conditions during ultrasonication treatment. These extreme physical conditions could be very high temperatures (up to 5000 °K), high pressures (up to 100 MPa), or shock waves [15,37].

### 3.2. Surface Hydrophobicity

**Figure 2:**
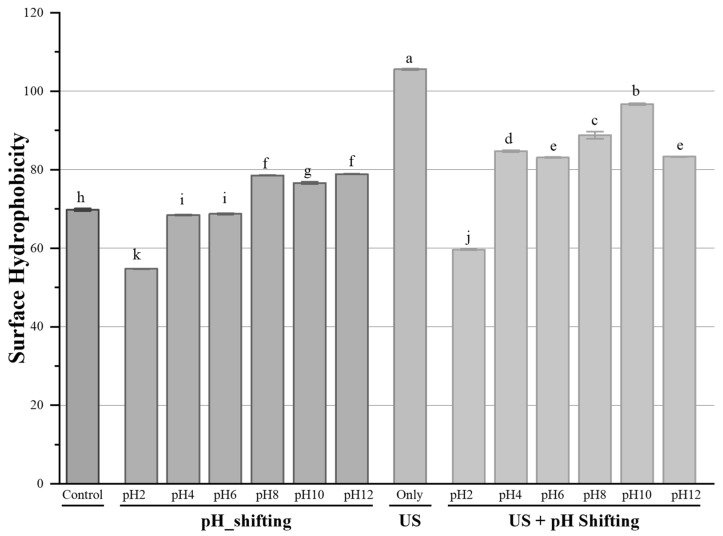
The surface hydrophobicity of non-treated and treated HPC samples. ^a–k^ Treatment means with the same letter in each sample are not significantly different (*p* > 0.05).

The interactions of hydrophobic parts have an important role in the solubility, stability, and functionality (foaming, emulsifying, gelling, etc.) of proteins [23]. The effect of different treatments on the surface hydrophobicity value of HPC dispersions is shown in Figure 2. Ultrasonication treatment resulted in a gradual but significant increase in the surface hydrophobicity when the ultrasonication combined with pH shifting treatment at different pH levels. The highest surface hydrophobicity was achieved at ultrasonication only treatment, which was 60% higher than the control. The pH sifting treatment alone at pH 2, 4, and 6 significantly decreased (*p* < 0.05) the surface hydrophobicity to 54.8, 68.6, and 68.9 from 70.1, respectively. Conversely, the pH shifting treatment alone increased the surface hydrophobicity at pH 8, 10, and 12 from 70.1 to 78.6, 76.9, and 78.8, respectively. Moreover, the combination of ultrasonication with pH shifting treatment at all levels except pH 2, resulted in a further increase of surface hydrophobicity. Parada et al. [38] evaluated the effect of ultrasonication with the frequency of 20 kHz at 20% and 40% amplitude for 0, 1, 3, and 5 min on a squid protein concentrate. In consistence with the present study, they found an upward trend in surface hydrophobicity as the time and amplitude were increased. In another study, Mao et al. [23] applied ultrasound at 20 kHz frequency and different power output levels (200, 400, 600, 800, and 950 W) for 60 min (2 s: 2 s off/on cycles) to cod protein samples and detected the similar trends in increasing ultrasonic power.

The increase in surface hydrophobicity might be because of the unfolding of protein structure after the pH shifting treatments. The ultrasonication application could further unfold the HPC, causing more exposure and relocation of hydrophobic groups at the surface of proteins [34,39,40]. The increase on surface hydrophobicity can facilitate a better formation of protein gel with more dense and compact properties in order to reinforce hydrophobic interactions [41].

### 3.3. Protein Quantification (Solubility) and Electrophoresis (SDS-PAGE)

Solubility is one of the most important key indicators of the physicochemical and functional properties of proteins [39,42]. Therefore, it has a crucial role in exploring the applicability of plant proteins in different industries [26]. The effects of different methods on the solubility of HPC are shown in Table 2. The solubility of HPC samples at pH 2, 4, and 6 significantly decreased (*p* < 0.05) compared to the control. This might be because the water interaction ability of the samples might be decreased at these pH levels due to the alterations in some polar compounds, such as carboxyl and amide groups [43]. Compared to the control, pH shifting treatment at pH 8 and pH 10 slightly increased the solubility of samples by 1.31% and 0.55%, respectively, although treatments with ultrasonication process at pH 8 and pH 10 significantly (*p* < 0.05) enhanced the solubility of HPC by 12.12% and 19.05%, respectively. This behavior was also pH dependent, and the effect of combined treatment was the lowest at pH 4 and pH 6 with a less increase in solubility. The reason might be the application of ultrasonication resulting in partial openings in the structure of proteins by increasing the surface area, facilitating more protein-water interactions and better solubility. Jiang et al. [44] also reported that an ultrasonication treatment applied at 20 kHz and 300 W for 12 min increased the solubility of black bean protein isolate compared to the untreated sample. Similarly, in the study of Huang et al. [42], the solubility of soybean protein isolate increased more than 156% over the control sample with a combined treatment of ultrasonication (20 kHz, 200 W, 10 min) and pH shifting (at pH 3) and reached to 80.9%.

#### Electrophoresis (SDS-PAGE)

**Figure 3:**
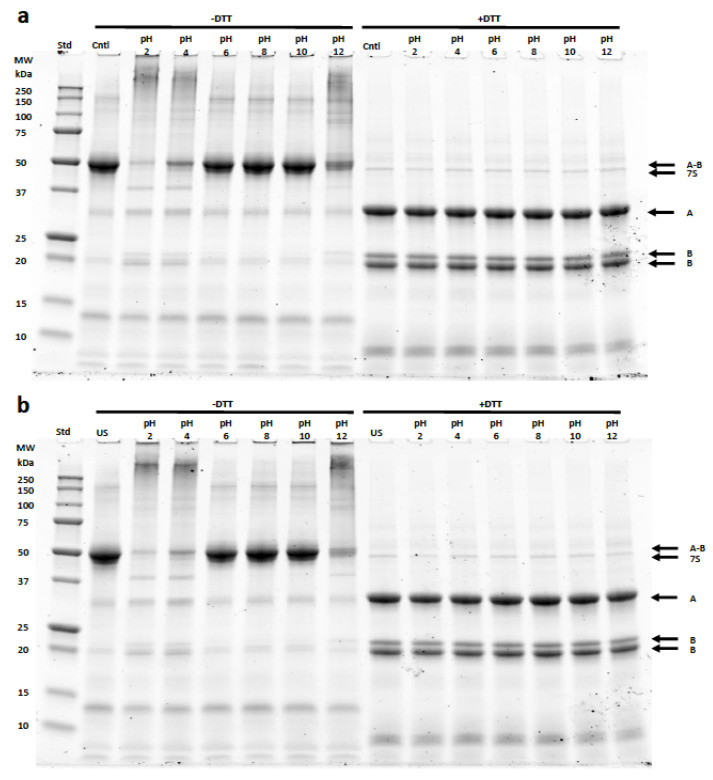
SDS-PAGE profile of HPC following pH shifting alone (**a**) and HPC following treatment with the combination of US with pH shifting (**b**). Samples were prepared with (+DTT) or without (−DTT). Std: molecular weight marker (kDa). HPC constituents: A/B (48 kDa) for Edestin; A, the acidic subunit (34 kDa), B, the basic subunit (20 and 188 kDa) for Edestin; 7S (46 kDa) for 7S globulin.

SDS-PAGE was used to analyze the HPC samples undergoing pH shifting, ultrasonication, and the combination of pH shifting and ultrasonication under non-reducing and reducing conditions (Figure 3). The identification of bands corresponding to the two major fractions found in hemp seed protein, albumins (~30%), and globulins (~60%), was based on previous studies [45,46]. The most prevalent protein found in the HPC is the globulin edestin which consists of six subunits with each subunit consisting of an acidic (Edestin A) and basic (Edestin B) component linked together by disulfide bonding. Specifically, bands below 18 kDa were attributed to albumins and bands of ~48 (Edestin A/B), ~46 (7S globulin), ~34 (Edestin A), ~20 (Edestin B), and ~18 (Edestin B) kDa, which were attributed to globulins. In all conditions, the albumins were preferentially solubilized over the globulins. pH shifting, especially at alkaline conditions, led to the aggregation of proteins shown by the increased staining intensity at higher molecular weights. The aggregation can be attributed to edestin due to the loss of staining intensity at 48 kDa under non-reducing conditions. 

The presence of reducing agent led to the disappearance of the higher molecular weight aggregates along with the appearance of the Edestin subunits A and B. The staining intensity of the Edestin A and B subunits was similar across all samples, further supporting that the initial aggregates at the acidic and alkaline conditions were related to Edestin. Similar results were reported by Wang et al. in 2018 in hemp protein isolate under alkaline conditions [46]. Under acidic conditions, there was the appearance of a band at 37 kDa. The band also appeared at pH 12, but with a lower intensity. Acid and alkaline conditions also lead to the dissociation of edestin by the increased intensity of bands at 34, 20, and 18 kDa. The addition of ultrasonication led to an increase in staining intensity above 250 kDa in samples shifted to pH 2, 4, and 12. The creation of aggregates by ultrasonication agrees with previous studies done with soy, hemp, pea, and potato protein [23,24,46]. Based on the solubility data, the increase in aggregation at pH 2, 4, and 12 led to an overall decrease in solubility compared to the control or ultrasonication only samples. Addition of ultrasonication led to an increase in the solubility of samples shifted to pH 8 and pH 10 over that of the pH shifted only samples. However, there is no significant difference in the electrophoretic profile between the samples shifted to pH 8 and pH 10 with or without ultrasonication.

### 3.4. Free Sulfhydryl Group Analysis

Free sulfhydryl content of proteins is one of the fundamental properties that affects the functional properties (gelling, emulsifying, foaming, etc.) of protein and it is an important index to evaluate the conformation change of proteins [20,47,48,49]. As shown in Table 2, compared with the control, the amount of free sulfhydryl content of HPC treated with only ultrasonication increased from 32.8 µmol/g to 40.68 µmol/g. Similarly, the free sulfhydryl content of ultrasonication assisted pH shifting treated samples at pH 6, 8, and 10 also increased significantly (*p* < 0.05) to 41.6, 58.72, and 46.54 µmol/g from 32.8 µmol/g, respectively. These samples also had a higher solubility than other samples, which shows that the increase in free sulfhydryl content contributed to better solubility. On the other hand, pH shifting only treatments showed that the concentration of the free sulfhydryl group increased gradually from pH 4 to pH 10. According to Jiang et al. [26] and Tan et al. [50], the increase in free sulfhydryl content might be associated with the increasing intramolecular disulfide bond cleavage reactions as the media becomes more alkaline. On the contrary, adjustment of the protein solution to pH 12 significantly reduced (*p* < 0.05) the free sulfhydryl content which might be due to the generation of disulfide bonds by sulfhydryl oxidation and interchange reactions between sulfhydryl and disulfide groups at extreme alkaline pH levels [26].

The results showed here concerning the free sulfhydryl content changes for ultrasonication assisted samples agreed well with those presented by Lee et al. [24], Malik et al. [16], and Xiong et al. [31], who also found a greater number of free sulfhydryl groups in sonicated samples of acidified soy protein isolate, sunflower protein isolate, and pea protein isolate. The acoustic cavitation causes breakdowns between the disulfide bonds and leads to an increase in free sulfhydryl content at the surface of the protein samples after ultrasonication treatments [48]. In contradiction to these studies, a significant decrease in free sulfhydryl groups by sonication treatments was also found on cod protein [51] and squid mantle proteins [52]. The reason of this decrease might be because of the oxidation of disulfide bonds with hydrogen peroxide molecules that formed by the generation of free radicals during sonication. This could explain the decreasing trend of ultrasound assisted pH shifting samples from 32.8 µmol/g to 28.65, 25.5, and 11.25 µmol/g at pH 2, 4, and 12, respectively.

### 3.5. Optical Characterization

The turbidity and color (L* (lightness), a* (redness), b* (yellowness), chroma, and hue angle) values of the samples treated with only ultrasonication, only pH shifting, ultrasonication with pH shifting, and non-treated were shown in Table 2 and Table 3, respectively. In general, the samples treated with only pH shifting had significantly higher L* values, and lower a* and b* values than the HPC samples treated with ultrasonication (*p* < 0.05). 

In accordance with the trends observed in color values, the turbidity of samples treated with pH shifting alone was the lowest among all samples. Turbidity could be defined as a function of the number and size of particles that dispersed in a dispersion [48]. Therefore, the number and size of soluble hemp protein aggregated in the mixture has a considerable effect on turbidity. In this study, the samples treated with the combination of ultrasonication and pH shifting had a significantly (*p* < 0.05) higher turbidity value than the samples treated with pH shifting alone. The collected data showed that the ultrasound applied samples had a higher solubility compared to other treated and control samples, which led the samples to have higher turbidity than others. This might be due to the disintegrating effect of ultrasound on soluble particles. Similarly, in the study of Jiang et al. [26], a significant increase in the turbidity values of commercial soy protein isolate was observed after being treated with sonication at the frequency of 20 kHz.

## 4. Conclusions

In the current work, the effects of ultrasonication (20 kHz) and pH shifting applications on the physicochemical and functional properties of HPC were investigated. Compared to ultrasonication and pH shifting alone treatments, their combination at alkaline pH levels worked synergistically and showed a significant improvement in the physicochemical and functional properties of HPC. The solubility of the samples was significantly improved by a combined treatment of pH shifting and ultrasonication with an increase from up to 1718.20 µg/mL from 1442.75 µg/mL. Additionally, the results indicated that the surface hydrophobicity of HPC could be enhanced almost 60% with the ultrasonication alone treatment. The particle size and polydispersity index of HPC dispersions also reduced significantly (*p* < 0.05) from 1.05 ± 0.05 mm to 0.33 ± 0.02 mm, and from 55.9 ± 1.2% to 12.1 ± 0.02% after the combination of pH shifting at pH 4 and ultrasonication treatment, respectively. The results of this study demonstrated that the application of ultrasonication and pH shifting combined treatment might be an effective alternative method for protein modification.

## Figures and Tables

**Table 1:** Hydrodynamic diameter, polydispersity index, and conductivity of non-treated and treated HPC samples.

	Hydrodynamic Diameter (µm)	Polydispersity Index (%)	Conductivity (mS/cm)
Control	1.048 ± 0.05 ^a^	55.90 ± 1.2 ^a^	0.958 ± 0.004 ^k^
pH2	0.945 ± 0.021 ^b^	46.20 ± 0.14 ^b^	1.143 ± 0.010 ^i^
pH4	0.920 ± 0.028 ^b^	42.90 ± 0.14 ^d^	1.302 ± 0.025 ^f^
pH6	0.925 ± 0.007 ^b^	46.80 ± 0.42 ^b^	1.210 ± 0.006 ^h^
pH8	0.905 ± 0.007 ^b^	42.30 ± 0.28 ^d^	1.025 ± 0.001 ^j^
pH10	0.915 ± 0.007 ^b^	40.80 ± 0.42 ^e^	1.272 ± 0.026 ^fg^
pH12	0.925 ± 0.007 ^b^	45.00 ± 0.28 ^c^	1.221 ± 0.016 ^gh^
US_Only	0.370 ± 0.014 ^def^	13.40 ± 0.14 ^g^	2.906 ± 0.021 ^b^
US + pH2	0.340 ± 0.014 ^ef^	16.35 ± 0.21 ^f^	2.186 ± 0.006 ^e^
US + pH4	0.33 ± 0.02 ^f^	12.10 ± 0.02 ^h^	2.275 ± 0.005 ^d^
US + pH6	0.400 ± 0.014 ^d^	16.30 ± 0.28 ^f^	2.329 ± 0.005 ^c^
US + pH8	0.380 ± 0.014 ^de^	14.30 ± 0.28 ^g^	2.962 ± 0.010 ^a^
US + pH10	0.425 ± 0.021 ^d^	16.95 ± 0.21 ^f^	2.203 ± 0.003 ^e^
US + pH12	0.510 ± 0.014 ^c^	17.30 ± 0.14 ^f^	2.169 ± 0.004 ^e^

^a–k^ Treatment means with the same letter in each sample are not significantly different (*p* > 0.05).

**Table 2:** Free S-S content, solubility, and turbidity of non-treated and treated HPC samples.

	Free S-S (umol/g)	Solubility (ug/mL)	Turbidity
Control	32.8 ± 0.212 ^f^	1442.75 ± 0.35 ^f^	0.222 ± 0.001 ^e^
pH2	24.45 ± 0.212 ^j^	1040.65 ± 0.21 ^n^	0.040 ± 0.001 ^k^
pH4	20.80 ± 0.424 ^k^	1312.65 ± 0.35 ^l^	0.052 ± 0.001 ^j^
pH6	27.70 ± 0.141 ^h^	1378.25 ± 0.35 ^h^	0.074 ± 0.000 ^i^
pH8	36.65 ± 0.212 ^e^	1462.10 ± 0.28 ^d^	0.146 ± 0.001 ^h^
pH10	42.35 ± 0.212 ^c^	1451.40 ± 0.14 ^a^	0.144 ± 0.001 ^h^
pH12	13.55 ± 0.071 ^l^	1069.35 ± 0.07 ^m^	0.196 ± 0.001 ^f^
US_Only	40.68 ± 0.042 ^d^	1432.90 ± 0.14 ^g^	0.321 ± 0.001 ^b^
US + pH2	28.65 ± 0.071 ^g^	1341.25 ± 0.35 ^k^	0.315 ± 0.001 ^b^
US + pH4	25.50 ± 0.141 ^i^	1544.15 ± 0.35 ^h^	0.227 ± 0.003 ^e^
US + pH6	41.61 ± 0.354 ^c^	1349.35 ± 0.35 ^j^	0.283 ± 0.002 ^d^
US + pH8	58.72 ± 0.028 ^a^	1617.70 ± 0.14 ^b^	0.301 ± 0.001 ^c^
US + pH10	46.54 ± 0.049 ^b^	1718.20 ± 0.28 ^e^	0.342 ± 0.001 ^a^
US + pH12	11.25 ± 0.354 ^m^	1372.30 ± 0.28 ^i^	0.176 ± 0.002 ^g^

^a–n^ Treatment means with the same letter in each sample are not significantly different (*p* > 0.05).

**Table 3:** Color values of non-treated and treated HPC samples.

	L*	a*	b*
Control	89.350 ± 0.78 ^e^	−0.060 ± 0.01 ^bc^	4.110 ± 0.01 ^a^
pH2	94.855 ± 0.01 ^b^	−0.275 ± 0.04 ^ef^	1.450 ± 0.06 ^g^
pH4	95.845 ± 0.05 ^a^	−0.245 ± 0.02 ^e^	0.920 ± 0.01 ^h^
pH6	93.130 ± 0.04 ^c^	−0.320 ± 0.01 ^fg^	2.405 ± 0.04 ^c^
pH8	93.655 ± 0.05 ^c^	−0.160 ± 0.01 ^d^	1.635 ± 0.04 ^f^
pH10	92.895 ± 0.02 ^c^	−0.020 ± 0.00 ^b^	3.135 ± 0.02 ^b^
pH12	92.015 ± 0.02 ^d^	−0.105 ± 0.01 ^cd^	3.160 ± 0.03 ^b^
US_Only	83.140 ± 0.06 ^h^	0.295 ± 0.02 ^a^	3.075 ± 0.04 ^b^
US + pH2	87.880 ± 0.04 ^f^	−0.440 ± 0.01 ^h^	1.770 ± 0.03 ^e^
US + pH4	88.385 ± 0.05 ^f^	−0.360 ± 0.01 ^g^	1.370 ± 0.03 ^gh^
US + pH6	86.120 ± 0.03 ^g^	−0.475 ± 0.01 ^h^	1.460 ± 0.03 ^g^
US + pH8	89.910 ± 0.04 ^e^	−0.675 ± 0.02 ^i^	1.280 ± 0.01 ^h^
US + pH10	82.740 ± 0.06 ^h^	−0.690 ± 0.01 ^i^	2.020 ± 0.01 ^d^
US + pH12	90.165 ± 0.09 ^e^	−0.160 ± 0.03 ^d^	1.265 ± 0.02 ^h^

^a–i^ Treatment means with the same letter in each sample are not significantly different (*p* > 0.05).

## Data Availability

Despite all information has been provided herein, raw data are available upon request to the corresponding author.
